# In Vivo Study of Osteochondral Defect Regeneration Using Innovative Composite Calcium Phosphate Biocement in a Sheep Model

**DOI:** 10.3390/ma14164471

**Published:** 2021-08-10

**Authors:** Lenka Kresakova, Jan Danko, Katarina Vdoviakova, Lubomir Medvecky, Zdenek Zert, Eva Petrovova, Maros Varga, Tatiana Spakovska, Jozef Pribula, Miroslav Gasparek, Maria Giretova, Radoslava Stulajterova, Filip Kolvek, Zuzana Andrejcakova, Veronika Simaiova, Marian Kadasi, Vladimir Vrabec, Teodor Toth, Vladimir Hura

**Affiliations:** 1 Department of Morphological Disciplines, University of Veterinary Medicine and Pharmacy in Kosice, Komenskeho 73, 041 81 Kosice, Slovakia; jan.danko@uvlf.sk (J.D.); katarina.vdoviakova@uvlf.sk (K.V.); eva.petrovova@uvlf.sk (E.P.); veronika.simaiova@uvlf.sk (V.S.); 2 Division of Functional and Hybrid Systems, Institute of Materials Research of SAS, Watsonova 47, 040 01 Kosice, Slovakia; lmedvecky@saske.sk (L.M.); mgiretova@saske.sk (M.G.); rstulajterova@saske.sk (R.S.); 3 Clinic of Horses, University of Veterinary Medicine and Pharmacy in Kosice, Komenskeho 73, 041 81 Kosice, Slovakia; zdenek.zert@uvlf.sk (Z.Z.); filip.kolvek@uvlf.sk (F.K.); vladimir.hura@uvlf.sk (V.H.); 4 Hospital AGEL Kosice-Saca, Lucna 57, 040 15 Kosice-Saca, Slovakia; maros.varga@nke.agel.sk (M.V.); tatiana.spakovska@nke.agel.sk (T.S.); jozef.pribula@agel.sk (J.P.); 5 Department of Engineering Science, University of Oxford, Parks Road, Oxford OX1 3PJ, UK; miroslav.gasparek@eng.ox.ac.uk; 6 Department of Biology and Physiology, University of Veterinary Medicine and Pharmacy in Kosice, Komenskeho 73, 041 81 Kosice, Slovakia; zuzana.andrejcakova@uvlf.sk; 7 Clinic of Ruminants, University of Veterinary Medicine and Pharmacy in Kosice, Komenskeho 73, 041 81 Kosice, Slovakia; marian.kadasi@uvlf.sk; 8 Clinic of Birds, Exotic and Free Living Animals, University of Veterinary Medicine and Pharmacy in Kosice, Komenskeho 73, 041 81 Kosice, Slovakia; vladimir.vrabec@uvlf.sk; 9 Department of Biomedical Engineering and Measurement, Faculty of Mechanical Engineering, Technical University of Kosice, Letna 9, 042 00 Kosice, Slovakia; teodor.toth@tuke.sk

**Keywords:** calcium phosphate biocement, cartilage, in vivo testing, sheep

## Abstract

This study aimed to clarify the therapeutic effect and regenerative potential of the novel, amino acids-enriched acellular biocement (CAL) based on calcium phosphate on osteochondral defects in sheep. Eighteen sheep were divided into three groups, the treated group (osteochondral defects filled with a CAL biomaterial), the treated group with a biocement without amino acids (C cement), and the untreated group (spontaneous healing). Cartilages of all three groups were compared with natural cartilage (negative control). After six months, sheep were evaluated by gross appearance, histological staining, immunohistochemical staining, histological scores, X-ray, micro-CT, and MRI. Treatment of osteochondral defects by CAL resulted in efficient articular cartilage regeneration, with a predominant structural and histological characteristic of hyaline cartilage, contrary to fibrocartilage, fibrous tissue or disordered mixed tissue on untreated defect (*p* < 0.001, modified O’Driscoll score). MRI results of treated defects showed well-integrated and regenerated cartilage with similar signal intensity, regularity of the articular surface, and cartilage thickness with respect to adjacent native cartilage. We have demonstrated that the use of new biocement represents an effective solution for the successful treatment of osteochondral defects in a sheep animal model, can induce an endogenous regeneration of cartilage in situ, and provides several benefits for the design of future therapies supporting osteochondral defect healing.

## 1. Introduction

Chondral injuries are a very frequent cause of pain and knee function limitation especially among active young adults and the working population. Articular cartilage is damaged mostly due to sports-related injuries, diseases, and trauma [1]. The treatment of these particular surface defects represents a substantial problem and has remained a difficult challenge for orthopaedic surgeons [2]. Patients with acute traumatic injuries of the joint have a higher risk of development of posttraumatic osteoarthritis, which results in severe pain and disability to perform activities of daily living, lower quality of life, and, eventually, total knee replacement procedures [3,4]. Within the past two decades, there have been many advanced surgical procedures for the treatment of traumatic or degenerative cartilage lesions. The principal limitation is the insufficient self-repair capacity of the hyaline articular cartilage because its avascular nature leads to insufficient nutrient supply to the damaged cartilage [1,5]. The superficial defects that only involve the articular cartilage do not heal spontaneously [6,7,8]. The osteochondral defects disrupt the structural integrity of the subchondral bone and gain access to the mesenchymal stem cells which contribute to cartilage regeneration [9,10,11,12]. Because of the very low repair potential and regenerative capabilities of hyaline cartilage, numerous treatment options for osteochondral defects exist. Current treatment modalities include osteochondral autograft transplantation, allograft chondrocyte implantation, autologous chondrocyte implantation, use of cells and molecules that promote chondrogenesis and inhibit cartilage breakdown, microfractures, subchondral drilling, marrow stimulation techniques, use of biodegradable scaffolds or various combinations of these [13,14,15]. Although multiple biomaterial strategies have been developed with the objective of repairing osteochondral tissue, there is currently no treatment available that would fully restore the function of the cartilage [16,17]. These methods can initially provide good clinical results such as pain relief and improvement of joint function, but each of these techniques still has its own limitations. Such methods often lead to formation of a fibrocartilagenous tissue of lower quality than the normal cartilage [14] or are not capable of stable regeneration of the cartilage tissue [18]. There is still no ideal material for defect filling, although a lot of studies have reported the successful use of various biomaterials [15]. A key requirement of osteochondral defect treatment is the ability to simultaneously repair both cartilage and bone in the osteochondral unit. The osteochondral unit is a complex system due to the architecture and function, with heterogeneity of components, e.g., cellular components, the presence of aggrecan, and collagen distribution. Primarily, the complexity of the osteochondral unit makes the approach to the setup of studies in regenerative medicine considerably demanding [19]. As a result, there is still a strong interest in new osteochondral defects treatment options.

Currently, significant attention is paid to biocements based on calcium phosphate due to their excellent biological properties. Calcium phosphate biomaterials were defined to be biocompatible [20,21]. Biocompatibility is characterised as a property of the biomaterial being compatible with living systems and tissues without inflammatory responses. Bioactivity of calcium phosphates is one of the most crucial biological features, for instance, cell adhesion, proliferation, adsorption of proteins, osteointegration and new bone formation [20,21,22,23]. To achieve these bioactive properties, biodegradation and ion release in biocements are essential [24,25]. The ion release has an impact on tissues, osteogenic cells, signalling pathways and various physiological processes. The increase in the calcium ions and phosphate ions concentration also promotes the formation of new bone minerals on the biocement surface and thus affects bone regeneration [20,26]. Osteoconductive features of calcium phosphate cements refers to the biomaterial capability and property that provides the bone growth on the biomaterial surface. Osteoconductivity is also crucial for bone regeneration. Calcium phosphates are considered to be osteoconductive because they allow the adhesion, proliferation, migration, and phenotypic expression of bone cells leading to the formation of new bone [27,28]. Osteoinduction is defined as an ability to induce and stimulate the progenitor cells to differentiate into osteoblastic lineages [20,29]. In general, calcium phosphates are osteoconductive but not osteoinductive. Nevertheless, some studies reported the ability of calcium phosphates to form bone tissue within nonosseous sites in vivo and without the supplementation of osteogenic factors [30].

We focused our research on the development of a biomaterial based on calcium phosphate biocement (CAL) with the addition of amino acids in acellular form, free of differentiation agents (e.g., growth factors). The amino acids used represent the main components in collagen (glycine, hydroxyproline, proline, and lysine), and we have hypothesized their positive impact on chondrogenesis and osteogenesis. Our study aimed to evaluate the effect of CAL on the healing of osteochondral defects and cartilage regeneration in the femoral trochlea of adult sheep. We have expected that autologous stem cells spontaneously differentiate into cartilage or bone, depending on the environment in which they are situated, and keep the original border between bone and cartilage. It was hypothesized that CAL applied to an osteochondral defect can induce and stimulate simultaneous regeneration of hyaline cartilage and subchondral bone.

## 2. Materials and Methods

### 2.1. Preparation of Biocements

Biocements were prepared according to Medvecky et al. [31]. Tetracalcium phosphate Ca_4_(PO_4_)_2_O was made by annealing process of an equimolar mixture of dicalcium phosphate anhydrous CaHPO_4_ (Fluka, Buchs, Switzerland) and calcium carbonate CaCO_3_ (Sigma-Aldrich, Saint Louis, MI, USA) for 5 h at the temperature 1450 °C. Subsequently, the tetracalcium phosphate was milled in a planetary ball mill (Fritsch, Idar-Oberstein, Germany, 730 rpm) for 2 h.

#### 2.1.1. C Cement

The cement mixture of tetracalcium/monetite powder was prepared by in situ reaction of tetracalcium phosphate and orthophosphoric acid (86%, Merc, Darmstadt, Germany) in 80% *v*/*v* ethanol by using a planetary ball mill with agate balls and vessel for half an hour. The orthophosphoric acid was added to the cement mixture in such quantities that the Ca/P mole ratio was 1.67.

#### 2.1.2. CAL Cement

The glycine: hydroxyproline: proline: lysine (CAL, mass ratio = 4:2:2:1) were dissolved in a reaction solution composed of orthophosphoric acid (86% analytical grade, Merck, Darmstadt, Germany) in 80 *v*/*v*% ethanol (reaction solution) and filtered through a 0.2 μm membrane filter (Millipore, PVDF, Darmstadt, Germany) in a laminar box (ESCO, class II, Esco Micro Pte Ltd., Singapore, Singapore); the sterile solution was then added to tetracalcium phosphate (synthesized according to Medvecky et al. [32], sterilized at 160 °C/2 h) and the suspension was milled in a planetary ball mill with agate balls and vessel for 30 min and dried at 100 °C under sterile conditions. The Ca/P mole ratio in cement was close to 1.67. Resulting CAL cement powder mixtures contained 4 wt% of amino acids. The cement paste was prepared by mixing the powder mixtures with 2% NaH_2_PO_4_ (as hardening liquid, sterile solution) at P/L ratio = 2. The quantities of components are listed in Table 1.

The final cement paste was prepared just before application to the osteochondral defect by mixing the C or CAL powder mixtures with 2% NaH_2_PO_4_ (as hardening liquid, sterile solution) at P/L ratio = 2. We used 1500 mg of powder and 750 µL of hardening liquid for complete filling of the one osteochondral defect. The hardening time of C and CAL cements was 5 ± 1 min.

The characteristic and properties of C and CAL cements can be found in our previous article [31]. In the microstructure of C cement, we found a low number of larger pores (10–15 µm) and a high fraction of 1–3 µm size micropores. In CAL cement, a few of the bigger macropores (10–20 µm) and high portion of 1–3 µm pores were found. The relative densities of C and CAL cements were 48.3 ± 0.3% and 43 ± 0.4%. The ph of SBF was at 7.7 after 24 h of C cement soaking, and no changes were found in the prolonged soaking time. The ph values during the CAL cement soaking were around 7.5 and did not change with soaking time. The compressive strength of C and CAL cements were 40 ± 3 MPa and 14 ± 0.7 MPa. Non-cytotoxic character of biocements was verified in vitro [31].

### 2.2. In Vivo Models for Regeneration of Osteochondral Defects and Surgical Technique

All animal procedures were approved by the State Veterinary and Food Administration of the Slovak Republic No. 2220/17-221. The study was realized on 18 healthy female sheep of the Valachian/Merino sheep breed. At the time of surgery, animals were from 2 to 2.5 years of age and from 60 to 75 kg in weight. General anaesthesia was achieved with butorphanol (0.1 mg/kg, Butomidor 10 mg/mL, Richter Pharma, Wels, Austria), and medetomidine 0.02 mg/kg (Cepetor 1 mg/mL, CP-Pharma Handelsgesellschaft, GmbH, Burgdorf, Germany) administered intramuscularly, and ketamine 8 mg/kg (Ketamidor 100 mg/mL, Richter Pharma, Wels, Austria) administered intravenously.

The sheep were divided into three groups: group 1 defects treated with the biomaterial CAL (*n* = 6), group 2—defects treated with the biomaterial C (*n* = 6), group 3—untreated empty defects left for the spontaneous healing (*n* = 6). Control samples (*n* = 6) were taken from the natural, original cartilage after removing the osteochondral stoppers from the area of created defects.

**Figure 1:**
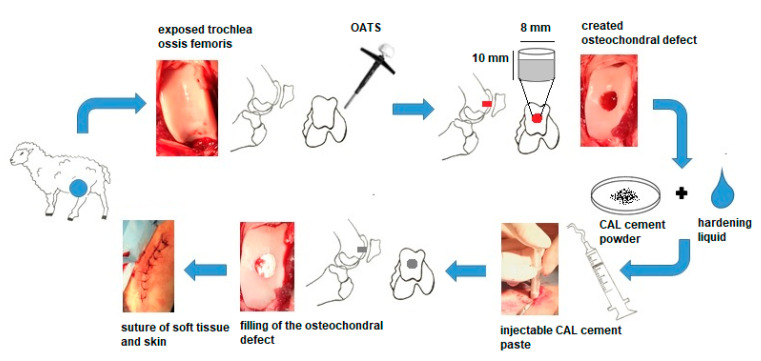
The scheme of the surgical procedure.

In all animals, the left hindlimb was shaved around the stifle joint and prepared with Betadine and alcohol using a sterile technique. To access the trochlea femoris, a 10 cm–long skin and soft tissue incision at the medial edge of the patella was performed. After arthrotomy, luxation of the patella was made, and the trochlea femoris was exposed. Standard osteochondral autograft transfer system (OATS) core punch (Arthrex, Naples, FL, USA) was used to create an osteochondral defect at a diameter of 8 mm and a depth of 10 mm in the trochlea ossis femoris (Figure 1). The long axis of the defect was perpendicular to the articular cartilage surface. OATS core punch allows creating defects with controlled diameter and depth. The lack of bone fractures, arthropathy, and correct orientation of the defects was validated by X-ray (Philips Digital Diagnost, Delft, The Netherland). Six removed stoppers, randomly selected, were used as control samples. In the treated groups, the created defects were filled with biomaterial C or CAL to the level of the adjacent cartilage. In the untreated group, the same defects were created, but without filling with the material. Immediately after the defect filling, the muscles, soft tissues, and skin were sutured, and the wound was covered by aluminium fluid spray.

### 2.3. Postoperative Management

After surgery, animals were returned to their cages and allowed to move freely with no external support. No problems with locomotion were noted in any of the animals during the 6-month follow-up period. There was no evidence of restriction of movement or any other negative effects of the treatment. As antibiotic prophylaxis, oxytetracycline dihydricum 20 mg/kg (Alamycin LA a.u.v., Norbrook, Newry, UK) was administered for 7 days after surgery intramuscularly, once every second day. Flunixin meglumine 2.2 mg/kg (Flunixin a.u.v., Norbrook, Newry, UK) was administered for immediate post-surgical pain management intramuscularly for 7 days (once a day) and then as needed for pain control. The sheep were euthanized after 6 months with Xylazine 0.2 mg/kg (Rometar 20 mg/mL inj., Bioveta) administered intramuscularly, and after 15 min Ketamin 2 mg kg (Narkamon 100 mg/mL inj., Bioveta) was administered intravenously. At the time of euthanasia, animals were from 64 to 82 kg in weight. The knee joints were examined by X-ray and MRI, subsequently were opened, the appearance and the quality of the filling were evaluated by gross analysis and histological analysis.

### 2.4. Gross Characteristic and Histological Analysis

The gross analysis included observing the general shape of the cartilage surface within the osteochondral defect, the area around it, the colour, consistency, smoothness, regularity, and integrity of the newly formed cartilage in comparison with the natural adjacent cartilage. The samples for histological analysis were obtained using the osteochondral autograft transfer system (Arthtrex, Naples, FL, USA). The osteochondral stoppers were fixed in neutral formalin, decalcified in chelation solution, dehydrated in series of ethanol, and finally, embedded in paraffin. Sections were done at 7 µm parallel to the long axis of the defect using a microtome (Leica, Bensheim, Germany) and stained according to standard protocols with hematoxylin-eosin to assess cell morphology, Safranin O and Alcian blue to detect proteoglycan accumulations and distribution in the extracellular matrix. The Picrosirius red and Masson’s trichrome staining were used to prove the presence of collagen. The type of collagen II and collagen I were detected immunohistochemically. We used a primary antibody Rabbit polyclonal anti collagen antibody (Abcam) for collagen II and Rabbit polyclonal antibody to collagen I (Biorbyt), and a secondary antibody in DB DET SYS kit (Biotech) according to the standard manufacturer’s instructions. Visualization of collagen II and collagen I was performed with DAB (3,3′-diaminobenzidine) (DAKO) substrate. Slides were viewed by the light microscope Olympus CX 43 (Olympus Corporation, Tokyo, Japan), and a 300 MIPromicam digital camera (Promicra, Prague, Czech Republic). Histological samples were evaluated by two observers and five samples for each staining were evaluated per osteochondral defect.

### 2.5. Microscopic Evaluations

Microscopic evaluations were performed using the Pineda histological scoring system [33] and modified O’Driscoll histological score [4].

#### 2.5.1. Pineda Histological Scoring System

The following parameters and points were evaluated: (A) the fulfilling of the defect: 125% (1 point), 100% (0), 75% (1), 50% (2), 25% (3), 0% (4); (B) the reconstruction of the osteochondral interface: yes (0), almost (1), not close (2); (C) the staining of the extracellular matrix: normal (0), reduced staining (1), significantly reduced staining (2), faint staining (3), no stain (4); (D) the cell morphology: normal (0), most hyaline and fibrocartilage (1), mostly fibrocartilage (2), some fibrocartilage, mostly nonchondrocytic cells (3), nonchondrocytic cells (4). A score of 0 indicates normal cartilage, and score of 14 indicating the most severe cartilage defects and no healing process [33].

#### 2.5.2. Modified O’Driscoll Scoring System

Number of points in individual categories according to modified O´Driscoll score [4].

(I) Nature of predominant tissue: hyaline cartilage (4), mostly hyaline cartilage (3), mixed hyaline and fibrocartilage (2), mostly fibrocartilage (1), some fibrocartilage, mostly nonchondrocytic cells (0); (II) structural characteristics: (A) surface irregularity: smooth and intact (3), superficial horizontal lamination (2), fissures (1), severe disruption, including fibrillation (0); (B) structural integrity, homogeneity: normal (2), slight disruption, including cyst (1), severe disintegration, disruptions (0); (C) thickness: 100% of normal adjacent cartilage (2), 50–100% or thicker than normal (1), 0–50% of normal cartilage (0); (D) bonding to adjacent cartilage: bonded at both ends of graft (2), bonded at one end or partially both ends (1), not bonded (0); (III) freedom from cellular ranges of degeneration: (A) hypocellularity: normal cellularity (2), slight hypocellularity (1), moderate hypocellularity or hypercellularity (0); (B) chondrocyte clustering: no clusters (2), ˂25% of the cells (1), 25–100% of the cells (0); (IV) freedom from degenerate ranges in adjacent cartilage: normal cellularity, no clusters, normal staining (3), normal cellularity, mild clusters, moderate staining (2), mild or moderate hypo/hypercellularity, slight staining (1), severe hypocellularity, poor or no staining (0); (V) subchondral bone: (A) reconstruction of subchondral bone: normal (3), reduced subchondral bone reconstruction (2), minimal subchondral bone reconstruction (1), no subchondral bone reconstruction (0); (B) inflammatory response in subchondral bone region: none/mild (2), moderate (1), severe (0); (VI) Safranin O staining: normal or near normal (3), moderate (2), slight (1), none (0). The maximum number of points is 28 points [4].

### 2.6. X-ray, Micro-CT, and MR Imaging

MR imaging was made with an MR imaging system at 1.2 T (Hitachi Oasis, Open system, Hitachi Medical Systems Holding AG, Tokyo, Japan). Scanning parameters: ET:10; TR: 3500–3805; TE:36.0; pixel size: 0.166; thickness of slices was 2–3.5 mm. MRI provides information on the physiological characteristics of the hyaline cartilage, and it is sensitive to discover chondral lesions. Further, MRI supplies information on the thickness of the cartilage, irregularities in the neoformed surface, morphological changes, anomalies of the subchondral bone and changes in signal intensity of the repair tissue. T1-weighted sequences were mainly used for the evaluation of the subchondral bone. T2-weighted sequences were used for characteristics and changes in the signal of the hyaline cartilage, and the interface between the cartilage, subchondral bone, and the synovial fluid. X-ray (Philips Digital Diagnost, Delft, Netherland) at 55–60 kV; 1.8–4.9 mAs; pixel size 0.133 mm was used for evaluation of potential osteoarthritis, subchondral cyst formations, or osteophytosis in all groups. After histological sampling, the presence of undegraded biomaterial in trabecular bone was observed in two cases (CAL biomaterial), and in three cases (C biomaterial). The integration of the remaining biomaterial with trabecular bone was evaluated by micro-CT (Metrotom CT, Carl Zeiss, Germany), and was compared to the neoformed trabecular bone in animals with resorbed and degraded biomaterial. CT scanning parameters: 140 kW; 350 µA; integration time 1000 ms; voxel 48.2 µm. CT images were analysed with the software VGStudioMax 2.2 (Volume Graphics, Germany).

### 2.7. Statistical Analysis

The histological results were assessed using One-way ANOVA with Tukey’s post hoc analysis (GraphPad Prism 5.0 for Windows, GraphPad Software, San Diego, CA, USA). All data are means with standard deviation of mean (SD). Average values (mean ± SD) within columns are statistically significant at the level of * = *p* < 0.05, ** = *p* < 0.01, *** = *p* < 0.001.

## 3. Results

### 3.1. Macroscopic Analysis of In Vivo Regenerated Articular Cartilage of Trochlea Humeri in Osteochondral Defects

**Figure 2:**
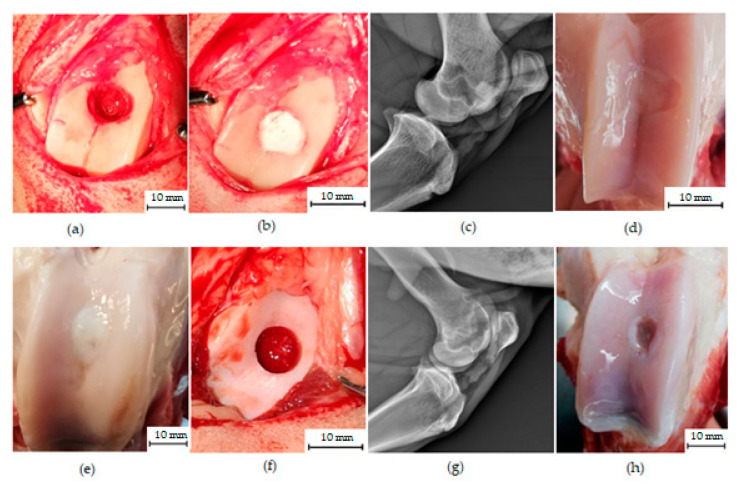
Gross appearance of the treated osteochondral defect (**a**–**e**) and untreated osteochondral defect (**f**–**h**) of the femoral trochlea in sheep. (**a**,**b**) The created defect of diameter 8 mm was filled by biomaterial CAL. (**c**) The absence of bone fractures and arthropathy was validated by X-ray. **(d**) At 6 months post-operation, the treated defect was covered by the consistent, smooth, glistening hyaline-like tissue indistinguishable from the adjacent native cartilage; however, the defect was still visible macroscopically and a small depression was present at the edge of the defect. (**e**) The defect treated with C biomaterial. (**f**–**h**) The same osteochondral defect was created in the untreated group. It was repaired with fiber-like tissue, leaving an irregular surface and a large depression in the centre of the defect. No macroscopic signs of inflammation were found within the joint cavities.

No signs of infection, inflammation or pathological changes in the group treated with biomaterial CAL were macroscopically observed. Within joints there were not found free remnants of cartilage or osteophyte formation. The synovial fluid was uniformly colourless and clear. The regenerated areas of defects were still visible in each examined knee, while the edge of the newly formed tissue was tightly connected to the adjacent native cartilage. There was a good integration between newly formed and native surrounding tissue. The treated osteochondral defects had visible signs of cartilage regeneration and had a better surface appearance and greater healing compared to untreated defects. Most of the treated defects were covered by smooth, continuous, hyaline-like tissue with colour identical or slightly whitish to the original cartilage. In two samples, a small hollow at the edge of the defect was visible, and in three cases, the surface of neocartilage was weakly concave (Figure 2).

In the group treated with C biocement, a slightly uneven surface was observed in three cases. In most cases, the colour of the healed defect was whitish and distinguishable from the native cartilage. The demarcation of the healed defect was more pronounced compared to the group treated with biocement CAL. No signs of pathological changes were macroscopically observed.

In contrast, the untreated samples showed remarkable cartilage damage and less evidence of cartilage regeneration. In most cases, the untreated osteochondral defects were partially repaired with fibre-like tissue, white or reddish in colour leaving an irregular surface with cracks and depressions at the defect site (Figure 2).

### 3.2. Histological Evaluation

#### 3.2.1. Treated Osteochondral Defects

**Figure 3:**
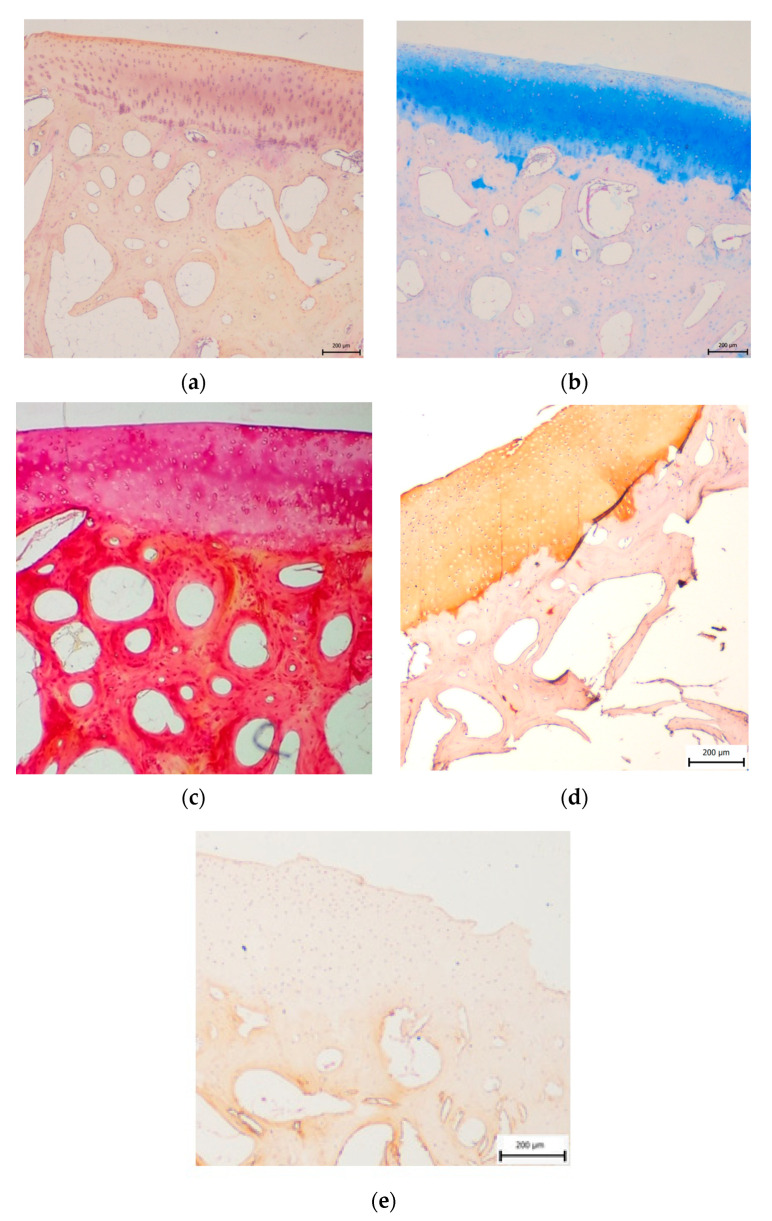
Control sample of hyaline cartilage and subchondral bone obtained from the native osteochondral stopper. (**a**) Haematoxylin-eosin staining, (**b**) Alcian blue staining, (**c**) Picrisirius red staining, (**d**) immunohistochemical evidence of collagen II, (**e**) immunohistochemical detection of collagen I; scale bar = 200 μm.

The structure of the neocartilage in the treated groups was comparable to the native hyaline cartilage (Figure 3).

**Figure 4:**
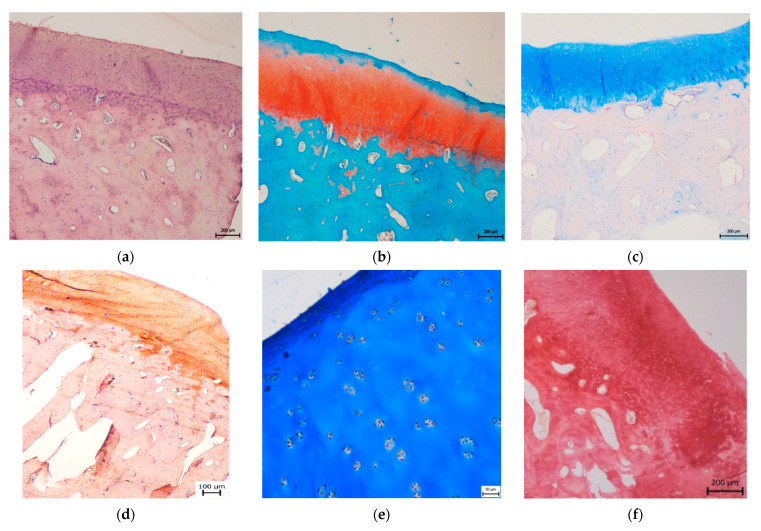

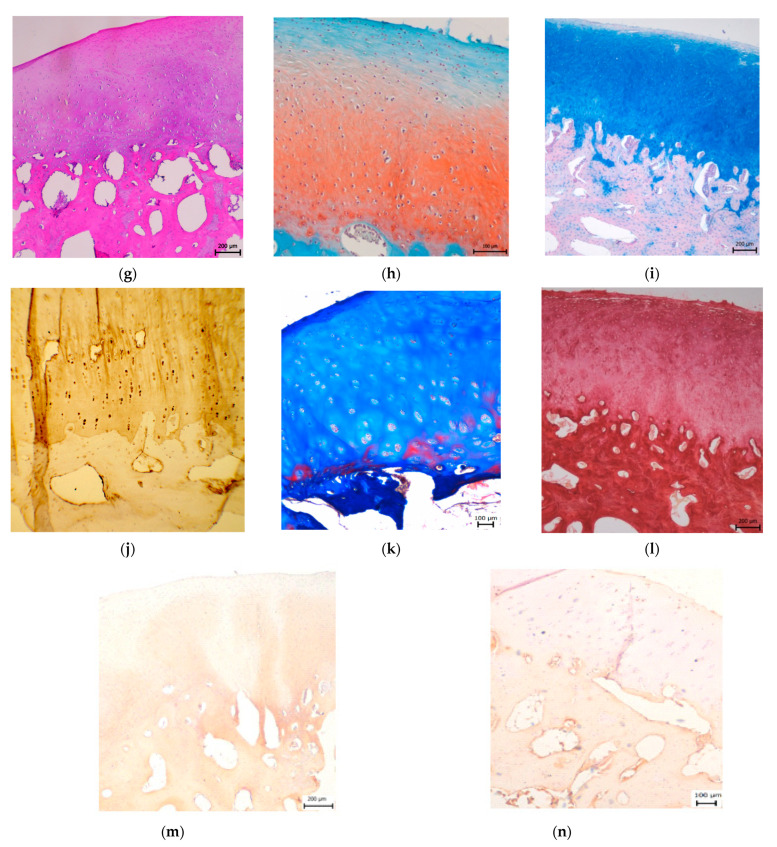
Histological evaluation of the articular cartilage regeneration in the treated group at 6 months post-operation. (**a**–**f**) The treatment with CAL biocement. (**a**) The formation of hyaline cartilage-like tissue, cartilage layers, and smooth surface as the native cartilage could be seen with Haematoxylin-eosin staining (scale bar 200 µm). (**b**) Safranin O staining and (**c**) Alcian blue staining showed similar stain intensity and morphological similarity to normal hyaline cartilage indicating a considerable amount of proteoglycans (scale bar 200 µm). (**d**) Immunohistochemical detection of type II collagen (scale bar 100 µm). Homogeneous distribution of collagen was detected by (**e**) Masson´s trichrome (scale bar 50 µm), and (**f**) Picrosirius red (scale bar 200 µm). (**g**–**l**). The treatment of C biocement. (**g**) Haematoxylin-eosin staining (scale bar 200 µm), (**h**) Safranin O staining (scale bar 100 µm), (**i**) Alcian blue staining (scale bar 200 µm), (**j**) immunohistochemical evidence of collagen II (100 µm), (**k**) Masson´s trichrome staining (scale bar 100 µm), (**l**) Picrosirius red staining (scale bar 200 µm). (**m**,**n**) immunohistochemical detection of collagen I, CAL treated defects, (**m**) scale bar 200 µm, (**n**) scale bar 100 µm.

In the CAL group, the cartilage morphology was predominantly hyaline, however, in three cases, the partial presence of fibrous tissue fibres was observed. All of the treated defects showed no signs of degenerative changes and demonstrated the formation of continuous hyaline cartilage layers. The superficial/tangential zone contained a relatively high number of cells with flattened morphology and parallel orientation to the cartilage surface. The matrix had a low content of proteoglycans which agree with the control samples. The chondrocytes in the middle zone were rounded and well distributed but sparsely present relative to the control samples. Moreover, the proteoglycan deposition reaches its maximum in the zone. In the deep/radial zone, chondrocytes were organized in columns placed perpendicular to the cartilage surface. Proteoglycan content, as well as the cell density, were lower than in the middle zone. The tidemark was single, intact and not crossed by blood vessels (Figure 4). The calcified zone below the tidemark formed the interface between bone and cartilage, and appropriately joined the cartilage to the bone. The structure of the subchondral bone was intact with corresponding bone porosity. No statistically significant differences were observed among all groups in subchondral bone reconstruction. All treated defects showed excellent integration between the calcified cartilage and subchondral bone. No differences were revealed in the case of the surface integrity, irregularity, and homogeneity between treated and native cartilage groups. On the other hand, significantly greater values were identified at the treated defects compared to untreated defects (*p* ˂ 0.05) in surface irregularity. In the CAL group, the cartilage thickness was identical to the adjacent native cartilage and no significant differences were observed compared to control samples. Morphology of chondrocytes and proteoglycan production were similar in neocartilage and surrounding cartilage. In the group treated with CAL, the morphology of chondrocytes corresponds to the hyaline cartilage, despite the mild hypocellularity and chondrocyte clustering were rarely observed in certain sections. The formation of cartilage-like tissue was verified by the positive Safranin O and Alcian blue staining, indicating a substantial amount of proteoglycans. The presence of collagen and its homogeneous distribution was proved by staining with Picrosírius red and Masson´s trichrome. The synthesis of type II collagen was detected immunohistochemically. Type II collagen was expressed in the pericellular matrix of chondrocytes as well as in the territorial and interterritorial matrix. The collagen I was not present or only low amounts of collagen I was observed (Figure 4).

In the group treated with biocement C, we observed an irregular uneven surface with depressions in three cases. Significantly smaller values were identified at treated defects compared to the control group (*p* ˂ 0.05). In one case, we monitored surface fibrillation, and in one case, there was a fine layer of fibrous connective tissue with the presence of blood vessels on the cartilage surface. The organization of individual cartilage layers corresponded to the previous description of defects treated with CAL biomaterials. However, in two cases, we noticed an unclear formation of individual cartilage zones, and in four cases we found a higher number of fibrous fibres. We recorded both hypocellular and hypercellular regions in neoformed tissue. There were also areas with chondrocyte clusters. The Safranin O and Alcian blue staining revealed medium to strong intensity, which proves variously high content of proteoglycans (Figure 4). Staining of Picrosirius red and Masson’s Trichrome was positive for collagen. We proved the presence of type II collagen immunohistochemically. The type II of collagen was expressed in pericelullar matrix of chondrocytes, as well as in the territorial and interterritorial matrix, but in four cases the reaction was weaker. The type I of collagen was observed in higher amounts compared to defects treated with CAL biocement.

#### 3.2.2. Untreated Osteochondral Defects

**Figure 5:**
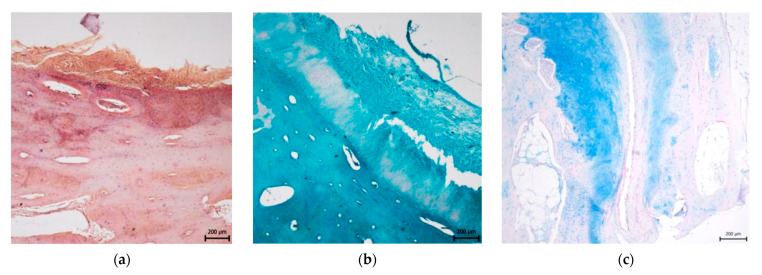

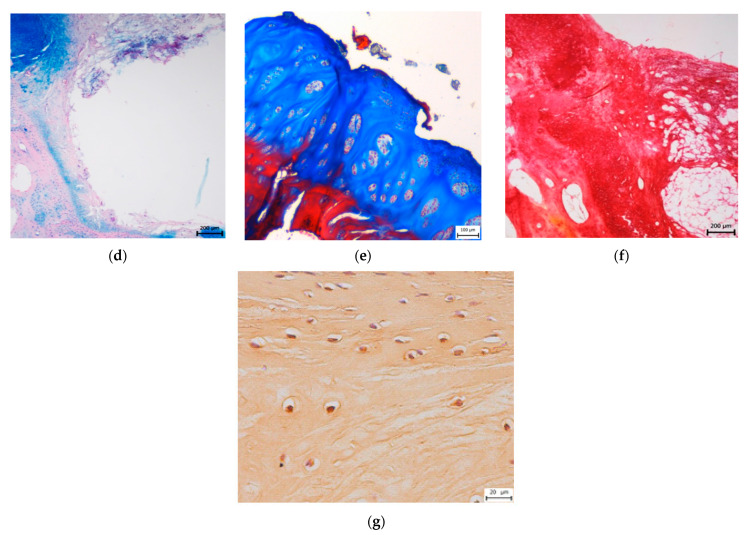
Histological evaluation of untreated defects left for spontaneous healing at 6 months post-operation. The untreated defect resulted in marked differences in cellular organization, mainly showing fibrocartilage, fibrous tissue, and disorganized tissue. (**a**) Hematoxylin-eosin staining showed no typical cell morphology and no cartilage tissue. The irregular surface was covered by a large quantity of fibrous tissue. (**b**) The defect showed almost negative Safranin O staining and chondrocyte clusters and hypocellular areas were found. (**c**,**d**) The deep fissure and decreased staining for Alcian blue were detected. The scale represents 200 µm. (**e**) The collagen distribution and chondrocyte cluster formation were observed, Masson´s trichrome (scale bar 100 µm). (**f**) Inhomogeneous tissue stained with Picrosirius red (scale bar 200 µm). (**g**) immunohistochemical staining of the type I collagen (scale bar 20 µm).

Contrary to the above facts, untreated spontaneous repair typically leads to the formation of fibrocartilage. The untreated defects had predominantly fibrocartilage morphology, except for two samples filled with collagen connective tissue or disordered tissue (Figure 5). The new tissues in untreated defects in most cases showed an irregular surface significantly different from the control samples (*p* ˂ 0.001). Statistically significant differences were also demonstrated between control samples and untreated defects in surface integrity and homogeneity (*p* ˂ 0.05). The deep cracks were in some cases extending into the calcified zone of cartilage or involving the full defect depth. In several cases, a fissure separated the neoformed fibrocartilage from the calcified zone close to the tidemark. There were significantly smaller values observed in the thickness of the newly formed tissue compared to control or CAL treated defects (*p* ˂ 0.05). The untreated defects showed a higher incidence of hypocellular areas, the presence of acellular zones alternating with hypercellular zones, and a more pronounced formation of the chondrocyte clusters. Chondrocyte clusters were characteristically round or oval and often localized near the clefts, fissures or close to areas filled with fibrous tissue. Safranin O staining and Alcian blue staining revealed variable changes in colour intensity and in comparison, with a control group, the statistically significant smaller values were recorded (*p* ˂ 0.001). An increase in the amount of type I collagen was observed in comparison with the amount of type II collagen.

**Figure 6:**
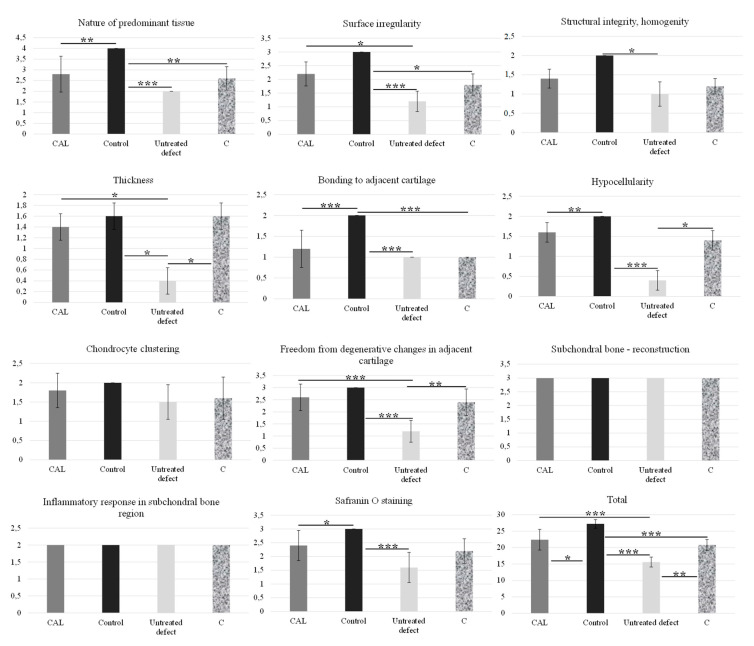
Results of modified O´Driscoll histological score. The total point means of the histological scores as well as most of the histological categories were significantly better in the treated group compared to the untreated group. Data are Mean ± SD. Average values within columns are statistically significant at the level of * = *p* < 0.05, ** = *p* < 0.01, *** = *p* < 0.001.

The modified O’Driscoll score and Pineda scoring system showed considerable differences in defect quality between the CAL group and the C group as well as between the CAL group and the untreated group (Table 2). According to modified O´Driscoll score [4], the measured histological scores were significantly higher in the CAL group than the untreated group (*p* ˂ 0.001). Moreover, a higher mean in the CAL group was found in most of the histological categories compared to untreated and C treated defects (Figure 6). The overall histological score Pineda et al. [33] shows significant differences between treated CAL group and untreated group (*p* ˂ 0.01), and between untreated group and control samples (*p* ˂ 0.001). These results confirmed that the application of CAL for the osteochondral defect treatment had enhanced osteochondral defect-repairing capabilities.

### 3.3. Magnetic Resonance Imaging Assessment of Cartilage Repair Tissue, Micro-CT and X-ray Evaluation

**Figure 7:**
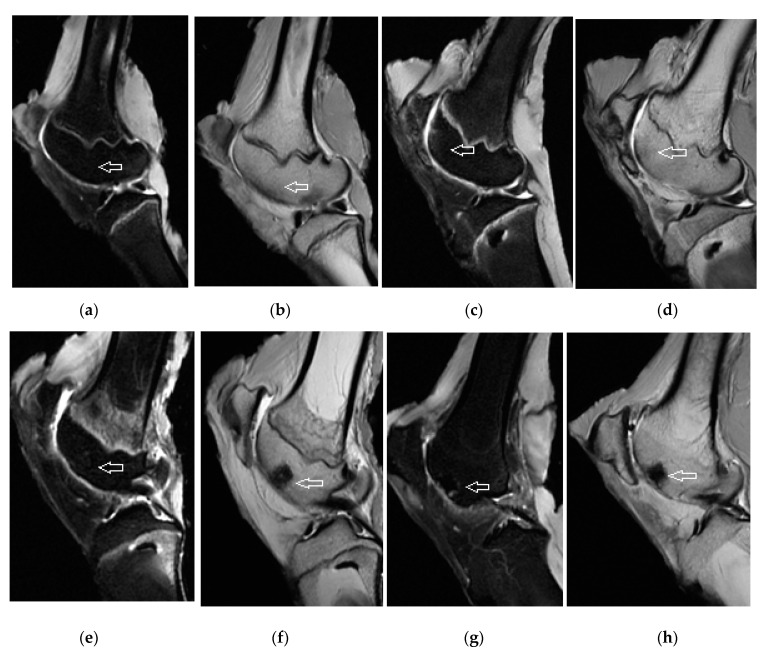
MRI of osteochondral defects treated with novel CAL biomaterial. (**a**–**d**) 6 months post-operation, MR images demonstrated complete defect filling with newly formed cartilage tissue at the level of surrounding cartilage or (**e,f**) slightly depressed, (**a**,**c**,**e**) with isointense signal and comparable thickness to native cartilage in T2-weighted fat-suppressed MR images. (**b**,**d**,**f**) The signal of trabecular bone in T1-weighted sequences was altered indicating bone remodelling with signs of subchondral sclerosis typical for the healing process. No signs of perifocal reactive bone oedema and no degenerative changes were observed. (**g**,**h**) MRI of osteochondral defect treated with C biocement. The thin margin of residual perifocal bone oedema was observed. The arrow indicates the area of healed osteochondral defect created in trochlea ossis femoris 6 months after surgery.

The quality of neocartilage formed in defects with CAL including tissue infill and surface continuity was much better compared to the cartilage in untreated controls. The results of MRI correspond to previous evaluations. The treated defects were filled with tissue that has a smooth, well-defined surface. The osteochondral defects were completely filled with the newly formed cartilage to the expected level equal to the adjacent articular cartilage. Three specimens were identified to be depressed. The neocartilage was of a comparable thickness to the adjacent cartilage. The architecture of the regenerated cartilage was homogenous, and it showed no cleft formations. The articular cartilage surface signals were isointense with surrounding native cartilage (Figure 7). By contrast, the untreated defects were incompletely filled with irregular inhomogeneous tissue. In most cases, the defects underfilling or slight hypertrophy leads to uneven articular contour. Hypointense or hyperintense areas were visible as well. The trabecular bones of treated defects were variously hyposignal in all sequences, indicating signs of bone remodelling with the characteristics typical for the healing process (Figure 7). The adjacent areas were without signs of perifocal reactive bone oedema. The CAL biomaterial did not induce specific degenerative changes in the joint cavity and in the adjacent native cartilage or bone. In addition, no synovitis was recorded in any of the scanned animals.

Osteochondral defects treated with C cement showed mild irregularity and presence of hypointense and hyperintense portions in three cases. Cartilage thickness was comparable to defects treated with CAL biomaterial and to surrounding healthy cartilage. In two cases, a thin margin of residual bone oedema was visible.

**Figure 8:**
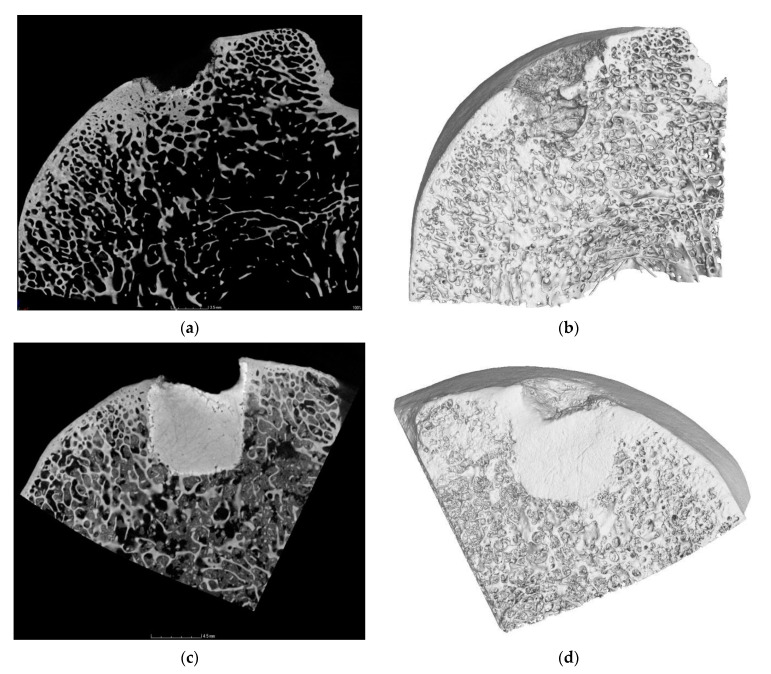
Micro-CT mapping of trochlea femoris showed (**a**,**b**) successful degradation of biomaterial and trabecular bone remodeling and regeneration. (**c**,**d**). Although insufficient biodegradability was observed in two cases. Completion of integration with the surrounding bone and there was no observation of pathological changes. Before the micro-CT, the samples of cartilage and subchondral plate were taken for histological evaluation. Sagittal sections, (**a**,**b**) scale bar = 3.5 mm; (**c**,**d**) 4.5 mm.

Micro-CT scans of the treated defects showed a much higher degree of bone regeneration and defect closure, associated with an increase within the bone-occupied areas, compared to the untreated defects. In most cases, the biomaterial was successfully degraded, subchondral and trabecular bone were regenerated. Although in two cases (CAL biomaterial) and in three cases (C biomaterial), the material has not been fully resorbed and degraded, integration of the biomaterial with adjacent trabecular bone was confirmed (Figure 8), and the materials were firmly attached to the bone. There were not any pathological changes, anomalous bone formation or bone penetrance into the cartilage. Conventional radiographs (X-ray) pointed to no joint space narrowing, subchondral cyst formations, osteonecrosis or osteophytosis in all groups. Similarly, no signs of osteoarthritis were depicted.

## 4. Discussion

For many years, researchers have been investigating the different scaffolds in the treatment of the osteochondral defect [34]. Complete repair of osteochondral defects to normal cartilage is rarely observed [1]. The most recent strategies for the treatment of osteochondral defects are based on the development of new biomaterials, which are able to induce regeneration of cartilage and subchondral bone, preferably through a one-step procedure in order to reduce risks for the patients and cost and to avoid manipulation with cells [35]. Calcium phosphate has been widely used in the field of bone regeneration in varied forms such as types of cement, coating, and scaffolds, based on its unique bioactive features, excellent biological properties, and bone regeneration efficiency [20,24]. Various studies have been carried out to improve the efficiency of calcium phosphate in combination with diverse healing agents [20]. A study by Kilian et al. [36] shows the successful use of a pasty calcium phosphate cement and a bioink based on alginate-methylcellulose in multi-layered mineralized constructs within 3D bioprinting of osteochondral tissue substitutes. Zhou et al. [37] reported bone defect repair properties of calcium phosphates/PLA modified tantalum scaffolds on the femoral trochlear groove of mature New Zealand white rabbits. Results indicate that the defective femoral trochlear grooves were well repaired with smooth surfaces and show that the scaffolds promote effective bone defect repair. A study by Vindas Bolanos et al. [38] investigated the effect of a composite scaffold with a calcium phosphate base for the repair of osteochondral defects in eight healthy horses. In the mentioned study, a cartilage-derived matrix combined with a three-dimensionally printed calcium phosphate cement was used to fill osteochondral defects in the femoropatellar joints. At the time of euthanasia, after 6 months of implantation, the surgical sites were readily recognizable by an indentation in the cartilage. Although the scaffolds appeared well integrated with the surrounding bone with good to very good adherence of the scaffold to the surrounding trabecular bone, in all cases Micro-CT analyses showed limited filling of the bony part of the osteochondral defect. Histological evaluation demonstrated that a predominantly fibrotic repair tissue had been formed. The neoformed tissue contained limited amounts of glycosaminoglycans. Positive Safranin-O staining was observed at the edges of the defect. A major part of the calcium phosphate cement had been resorbed and replaced by newly formed bone; remaining fragments were still visible.

Interestingly, it has been declared that several ions have a considerable role in neoangiogenesis. Silicon is a substantial element that has the ability to induce angiogenesis in the formation of new bone and it is able to increase the regeneration of bone tissue [39]. The study of Forni et al. [40] stated that the inclusion of calcium silicates-based materials to calcium phosphates, e.g., dicalcium phosphate dihydrate offers an interesting advantage. The addition of calcium silicate-based materials is manifested by an increase in biological properties of calcium phosphates and shows improvement of apatite forming abilities [41,42]. Calcium silicate biomaterials also point to biointeractive properties [41], ion release, and the induction of the cells’ differentiation, e.g., mesenchymal stem cells differentiations [43].

In the present study, it was demonstrated that treatment of osteochondral defects with acellular CAL biomaterial resulted in excellent subchondral bone and cartilage regeneration, with the characteristic structure of hyaline cartilage. Newly formed tissues were analysed after 6 months of healing of artificial osteochondral defects in sheep, which was a clinically appropriate large animal model from the point of view of osteochondral defect healing analysis [4]. As presented, the surfaces of the osteochondral defects were significantly better regenerated in the sites treated with CAL compared to the untreated sites, and considerably better regenerated compared to the sites treated with C biomaterial. The surface of the treated defects appeared filled and smoothly covered with hyaline-like tissue. There was a substantial difference for newly formed cartilage colour, and also the boundary to the surrounding adjacent cartilage was more unclear at the treated sites compared to the untreated sites. All mean microscopic scores regarding cartilage formation were also higher at the treated sites than at the untreated sites. The matrix in the regenerated tissue was homogeneously distributed, Safranin-O staining and Alcian blue staining as indicators of accumulation of proteoglycans in the regenerated tissue were more intense in the treated defects than the untreated group as well as the cellular morphology was comparable with the control sample. In the case of untreated defects, the reduction in proteoglycan content or no presence of proteoglycans were observed. The cracks were developed with distance from the superficial surface of untreated defects simultaneously with the decrease in the proteoglycan concentration. The scores of structural integrity and bonding to the adjacent cartilage were also significantly higher in the treated osteochondral defects.

Our results showed that an enhanced concentration of extracellular collagen amino acids had a positive effect on the healing process. Used amino acids, glycine, hydroxyproline and proline, provide 57% of the total amount of amino acids in collagen and they play a substantial role in collagen structure. They have important physiological and metabolic roles. Glycine is the main substance of all types of collagen [44,45]. Glycine has a great impact on chondrogenesis, and significantly increased accumulation of calcium, increased mRNA expression of osteopontin and alcaline phosphatase activity. Particularly, glycine could play a role in the mechanism of cartilage calcification [44,45]. The study of the foetal tibia in a mouse shows that the cartilaginous primordia significantly grew up in the presence of glycine [46]. Lysine has a positive effect on osteoblast activation, proliferation, and differentiation [47], and it has an important role in the crosslinking of the collagen fibres [48]. According to de Pas-Lugo et al. [45] culture medium enriched by lysine, glycine and proline improved the production of collagen II by chondrocytes.

In the study of Bernstein et al. [34], the modified O’Driscoll score showed significantly greater values in defect quality between the group treated with calcium phosphate ceramic scaffolds seeded with chondrocytes and the control group for the 26-week time point in sheep. Differences between the two groups were strongest for the cluster formation. None of the treated defects had more than 25% of cells involved in cluster formation, but over half of the cells were in clusters in the untreated defects. The total modified O’Driscoll score [4] 26 weeks after implantation was 14.42 ± 2.44 for treated defects and 11.85 ± 5.11 for untreated defects [34]. In comparison, our study showed higher total point means of the histological scores for CAL treated defects 22.4 ± 3.1, C treated defects 20.8 ± 1.6, and also for untreated defects 15.6 ± 1.5 (mean ± SD).

The important finding is the presence of the tidemark [49]. Pilichi et al. [12] reported that this calcification front is in a state of dynamic equilibrium, where factors supporting mineralization are balanced by substances that limit or inhibit the range of calcification. This process of active calcification and subsequent ossification appears integral to the joint shape and, therefore, to the distribution of load [12]. In our study, the tidemark was present, and the regenerated tissue was closely integrated with the native cartilage tissue and bone below it. New bone formation was also evident. Histologically, there was no observation of differences among all groups in the reconstruction of subchondral bone and inflammatory response in the subchondral bone region.

Several studies focused on improvement of biological properties of biomaterials using exosome enriched strategies. Exosomes are extracellular vesicles derived from mesenchymal stem cells which encourage cell communication [50]. Exosomes are mediators in cell communication, and they can modify the activity of target cells. They are involved in physiological processes such as neoangiogenesis, cell differentiation and proliferation, extracellular matrix remodelling, tissue homeostasis, and could deliver different informational molecules [51]. The study of Gandolfi et al. [50] positively assessed the addition of exosomes to scaffolds based on polylactic acid, dicalcium phosphate dihydrate and calcium silicates. The significance of exosome therapies lies in a novel approach to support bone regeneration. The strategy supports migration and proliferation of cells, angiogenesis and osteogenesis within the bone tissue formation. Exosomes can induce and regulate the mesenchymal stem cells to the osteoblastic lineage [52], and affect osteoblast activity and function.

In vivo study of Furuta et al. [53] points to the acceleration of femur fracture healing using exosomes in a mouse model. Improved bone regeneration and pro-osteogenic potential of exosomes combined with tricalcium phosphate scaffolds was observed in the study of Zhang et al. [54]. The excellent therapeutic effects of exosomes derived from human embryonic mesenchymal stem cells was also demonstrated on regeneration of osteochondral defects in a rat model [55]. The osteochondral defects were created on the trochlea ossis femoris. The exosome treated osteochondral defects showed complete regeneration of hyaline cartilage and subchondral bone, full integration with surrounding bone, good regularity of cartilage surface, high amount of glycosaminoglycans and collagen II, and low amount of collagen I. [55]. The treatment of bone and cartilage defects using exosome appears to be effective, has a high regenerative potential, and represents a cell free therapeutic alternative to stem cells therapy [51].

According to Sahin et al. [56], in order to achieve optimum clinical results, an adequate resorption rate is an important parameter that may vary with the clinical applications. The micropores in calcium phosphate cement do not allow rapid bone ingrowth, and osteoclastic cells are able to demote the hardened cement layer by layer only, beginning at the cement bone interface. Bone substitution rate depends on age, anatomic site, sex, and general metabolic health also. Considering these circumstances, it may take 3–36 months for the cement to be fully replaced by bone and resorbed [56]. However, in our study, the biomaterial degradation was not observed in two cases (CAL treated defects), or three cases (C treated defects), well integration of biomaterial with surrounding bone was noticed in both groups. Nevertheless, that we found signs of insufficient degradability of the material, it was associated with a continuous subchondral bone formation and also hyaline-like cartilage formation with the presence of all cartilage layers, although fine fibrous fibres in the cartilage matrix were observed. In accordance with histological findings, the results of micro-CT confirmed no pathological processes, new subchondral bone formation and strong integration of non-degraded biomaterial to the adjacent bone.

Some studies compare histological evaluation and micro-CT evaluation. The study of Lacourt et al. [57] correlated micro-CT assessment of subchondral bone structure with histological analysis in the carpal bone of equine athletes in different stages of osteoarthritis. Using a combination of micro-CT and histological evaluation, microstructural changes were observed in the calcified cartilage and underlying subchondral bone, e.g., calcified cartilage cracks, focal pits extended through the calcified cartilage and underlying subchondral bone. The subchondral bone changes monitored by micro-CT correlated with the histological lesions. The Study of Clark et al. [58] examined the utility of a standard laboratory micro-CT scanner to visualise and quantify features of the chondrocyte population in healthy cartilage. Histological staining was used to confirm these cartilaginous features observed by micro-CT. The chondrocytes density measured with micro-CT provided values within the range of chondrocytes and their lacunae measured with histological images. Morphology was compared with cellular roundness, and both techniques presented similar values.

The biocement applied in this study has several benefits due to its character, structural and physicochemical properties. It has biocompatible, bioactive, osteoconductive, biodegradable, and bioresorbable properties. The biocement paste is injectable and when administered into the osteochondral defect allows complete defect filling regardless of the defect shape. Several authors stated that precise positioning of the plug in the drill hole of the defect was crucial for the successful restoration of articular cartilage [34]. The administration form of the cartilage repair product is important for successful defect treatment. The CAL biomaterial helps in overcoming difficulties in the adhesion of biomaterials with the adjacent tissue and provides fulfilment with the surrounding cartilage and subchondral bone tissue. At the same, CAL represents one type of fast setting biocements that safely eliminates the removal of material by blood and provides appropriate mechanical support to ensure all physiological processes in vivo. Further, it supported good infiltration, differentiation, and proliferation of the mesenchymal progenitor cells. According to Sahin et al. [56], calcium phosphate cements represent the most complex surfaces that usually evoke a favourable cellular response for tissue regeneration. Their composition may ensure an effective chemical gradient to induce required cellular activity that leads to rapid wound regeneration. These are the main reasons for cement systems to function well in the body, mostly as hard tissue replacements [56].

## 5. Conclusions

In conclusion, the results of this study demonstrate that the treatment of osteochondral defects using innovative calcium phosphate biocement enriched by amino acids can induce simultaneous bone and cartilage formation with major signs of hyaline tissue in a large animal model. Excellent biocompatibility, osteogenic activity, and regenerative abilities of biocement used could also be attractive for clinical application in humans.

## Figures and Tables

**Table 1:** Quantities of various components in 1000 mg of CAL cement powder.

Components of CAL Cement	Quantity (mg)
TTCP/monetit	960/equimolar mixture
Aminoacids mixture:	40
glycine	17.8
hydroxyproline	8.9
proline	8.9
lysine	4.4

TTCP—tetracalcium phosphate.

**Table 2:** The total mean histological scores (mean ± SD).

Scoring Systems	Treated Group		Control	Untreated Group
	CAL	C		-
modified O’Driscoll	22.4 ± 3.1	20.8 ± 1.6	27.2 ± 1.3	15.6 ± 1.5
Pineda	1 ± 1	1.6 ± 0.5	0	3 ± 0.7

## Data Availability

Data are contained within the article.
